# Objectively Measured Physical Activity and Its Association with Functional Independence, Quality of Life and In-Hospital Course of Recovery in Elderly Patients with Proximal Femur Fractures: A Prospective Cohort Study

**DOI:** 10.1155/2020/5907652

**Published:** 2020-01-27

**Authors:** Laureen V. Marsault, Jesper Ryg, Carsten Fladmose Madsen, Anders Holsgaard-Larsen, Jens Lauritsen, Hagen Schmal

**Affiliations:** ^1^Department of Orthopedics and Traumatology, Odense University Hospital, Odense, Denmark; ^2^Department of Geriatric Medicine, Odense University Hospital, Odense, Denmark; ^3^Department of Clinical Research, University of Southern Denmark, Odense, Denmark; ^4^OPEN, Odense Patient Data Explorative Network, Odense University Hospital, Odense, Denmark; ^5^Clinic of Orthopaedic Surgery, Medical Center—University of Freiburg, Faculty of Medicine, University of Freiburg, Germany

## Abstract

**Background:**

Physical activity in elderly patients is crucial for recovery from proximal femoral fractures. Considering the limited possibilities for objective measurement, we aimed to evaluate the use of an accelerometer in this population to determine activity's association with functional independence, quality of life, and course of recovery.

**Methods:**

52 patients undergoing operative treatment for proximal femur fractures (81.3 ± 7.5 years) were included in a prospective cohort study. 12 patients with fall but without fracture of the lower extremities (80.8 ± 9.5 years) served as control. An Axivity AX3 tracker continuously recorded signal vector magnitudes during the hospital stay. Additionally, 2 ± 1 and 8 ± 3 days (time point 1 and 2) after operation EuroQol-5D and Barthel-20 indices were evaluated.

**Results:**

Physical activity increased in all patients with time. Multiple regression analyses revealed that a high Barthel-20 before fracture, a low age, a high body mass index, high albumin, and low C-reactive protein levels were independent predictors for high physical activity at time point 1 (*p* < 0.05). Physical activity correlated significantly with EuroQol-5D and Barthel-20 at time point 1 and 2 (*p* < 0.02). Furthermore, physical activity at time point 1 predicted EuroQol-5D, physical activity, and Barthel-20 at time point 2 (*p* < 0.01). A multiple regression demonstrated equal physical activity in patients with or without a hip fracture.

**Conclusions:**

Accelerometer signals correlate with postoperative physical activity, Barthel-20 and quality of life in elderly patients. Physical activity is thereby positively influenced by a high prefall functional independence and a good nutrition status. A timely and adequate operation provided, there is no difference between patients with or without a fracture. This trial is registered with DRKS 00011934 on 10^th^ April 2017.

## 1. Background

Proximal femoral fractures are common in older patients and are associated with a high mortality of around 25–30% within the first year [1]. This is determined by a variety of factors such as comorbidity and age, which cannot or only partially be altered [2]. An influenceable key factor for a successful reintegration into normal life is the recovery of mobility. Although most hip fracture patients do not regain their habitual physical activity (PA) level [3], an early start of rehabilitation and increased postoperative activity are associated with improved outcomes [4].

Unfortunately, an increased age, as well as hospitalization, is often accompanied by a decrease of cognitive function, which makes it difficult to assess a valid health and physical status of these older patients. Therefore, typical questionnaires such as the Oswestry Disability Index [5] or the Euroqol 5D [6] have limited capacities to evaluate this population. Activity trackers, also known as step counters, wearables or accelerometers, have recently obtained growing interest for surveillance of PA and were successfully validated in young people [7]. They were found to be reliable tools for PA measurements [8–11], although differences between various manufacturers were found [12]. The level of PA is very different comparing young and active with older people [13]. Until now, valid data for older adults are limited [14–16]. However, data evaluating activity in elderly patients after operations in association with function and quality of life are even only anecdotally available [17, 18]. Since clinical trials in older people need objective evaluation parameters, which can easily be obtained and are not dependent on active cooperation, accelerometers appear to be ideal tools for this special population. We hypothesized that accelerometers can monitor activity in older people and supply personalized information about the degree of recovery evaluated by scores for functional independence and quality of life. By this, two separate constructs are being measured and compared, patients' functional capacity (what they CAN do) vs. their actual activity (what they ACTUALLY do). Our study is based on a reliable accelerometer (Axivity AX3) [19] and recently validated algorithms for measurement of PA in this fragile population [20].

## 2. Methods

### 2.1. Study Design and Research Questions

The following research questions were analyzed by means of a prospective cohort study with aims and outcome measures registered prior to inclusion of patients:

- Which parameters influence activity?
- Are the activity measurements comparable to the Barthel-20 index and the EQ5D questionnaire?
- Can activity predict the recovery process of the patients?
- Are there any differences between hip fracture patients and geriatric patients admitted after a fall without a lower extremity fracture?

The present study conforms to the reporting format suggested by the STROBE panel [21].

### 2.2. Participants

Participants were recruited at the Odense University Hospital (OUH), Denmark, from 1^st^ March, 2017 until ultimo September 2017. A group of patients suffering from proximal femur fractures due to a fall was compared to a group of patients sustaining a fall without fractures of the lower extremities. The inclusion criteria were patients with a fall and acute proximal femur fracture (location 31 according to Arbeitsgemeinschaft für Osteosynthesefragen—AO) [22] (hip fracture group) or patients with a fall and fractures of the upper extremity or a fall within a week without a fracture, but necessity of hospital admission (group without hip fracture or fall group), being able to read and understand Danish, and age ≥65 years.

Exclusion criteria were open fractures, polytrauma, known colonization with multiresistant bacteria, no independent walking function before admission (bedridden patients), infection of the wound, operative revisions for other reasons, or Orientation-Memory-Concentration (OMC) test score <8.

### 2.3. Outcome Measurements and Registered Parameters

Physical activity was measured as recently described [20]. Briefly, patients were tracked continuously by the skin-taped Axivity™ AX3 tracker (Newcastle upon Tyne, UK), which was placed at the nonoperated antero-lateral femur. If patients were discharged before the 6^th^ postoperative day, they were asked to keep wearing the trackers and to send them back by postal service. The participants were blinded to their activity data. The Axivity AX3, a 3-axis accelerometer, provided the possibility to analyze raw accelerometer data based on the calculated signal vector magnitudes (SVM) using the following equation $abs\left(\sqrt{\left(x^{2}+y^{2}+z^{2}\right)}-1\right)$. Data were recorded in 60 second epochs applying a filter between 0.5 and 20 Hz and subjected to a wear time analysis after continuous data registration. Nonwearing intervals were excluded from analysis. The tracker was subsequently connected to a personal computer using an USB-interface. The analysis was done by the standard software (The [AX3] OMGUI Configuration and Analysis Tool, https://github.com/digitalinteraction/openmovement/wiki/AX3-GUI). Each minute was categorized into “active” or “not active” using a threshold of SVM > 0.005. Furthermore, 10-minute periods were categorized as no activity with 0–10% active minutes, low activity >10–25% active minutes, medium activity >25–60% active minutes, and high activity >60% active minutes. Active minutes with a threshold >0.01 were categorized as very high active minutes. The absolute numbers of active and very active minutes were analyzed and their relative portions within a 1440 minutes period (1 day).

The time points (TP) were defined as follows: the baseline data refer to the situation before admission and treatment. TP1 included day 1–3 after operation or admission to the department, if no operation was required. TP2 included day 5–11 and was recorded as close to the actual discharge as possible. Activity data reflect the average of all days within the applicable period.

The following questionnaires were evaluated: the Danish Barthel-20 index (Barthel-20)—a classical instrument for functional independence, the Danish European Quality of life 5 Dimensions 3 Levels (EQ5D-3L)—a classical instrument for quality of life and the Orientation Memory Concentration (OMC) test [23]. We also aimed to register the time from fall to admission or operation. Furthermore the participants' nutritional status was assessed with the questions: “Have you lost weight in the last 6 months” (current weight loss) and “Have you eaten less than usual in the past week” (eating reduction), which combined with the body mass index (BMI) and severity of the illness gives the Nutritional Risk Screening (NRS-2002) score [24, 25]. As a proxy for prefall frailty, a retrospective one repetition Sit-to-Stand test (STS) [26, 27] was acquired by asking the participants “Do you use your hands for support when you rise from a chair.” A preadmission mobility category (premobility) was assessed for each patient; no walking ability (bedridden) (corresponds to 1), need for aiding devices (corresponds to 2) or walking without help (corresponds to 3). The participants were also asked about their pain levels, using the Verbal Rating Scale (VRS), where 0 was best and 10 worst. Complications (e.g. infections, bleeding, secondary dislocations, secondary fractures) were registered at discharge, and analyzed binarily (yes or no). Hemoglobin, albumin, and C-Reactive Protein (CRP) levels were also registered, as they could influence activity/mobility. The patient characteristics, treatment characteristics, time from fall to admission, “Sit-to-Stand” (STS) index, cognitive status (OMC), nutrition status, and complications were registered once. The scores (EQ5D-3L and Barthel-20) and pain levels (VRS) were monitored twice during the hospital stay—at TP 1 and 2. Barthel-20 was also recorded retrospectively at baseline to determine the preadmission functional independence level. Serum levels of C-reactive protein (CRP), albumin, and hemoglobin were registered at admission (baseline), TP 1 and 2.

### 2.4. Statistics

REDCap™ (Research Electronic Data Capture) facilitated the data management. It is a database especially designed for biomedical research and fulfills all necessary safety features and is supported by the OPEN initiative (Odense Patient data Explorative Network).

Based on the preliminary results of the pilot study [20] we could assume a clinical relevant 25% increased activity from TP 1 to TP 2. Taking this and 20% dropouts into account, an 80% power for paired statistical analysis required the inclusion of 48 hip fracture patients to reach statistical significance. A second calculation for an unpaired group comparison between hip fracture patients and the group without a fracture of the lower extremities was then carried out. For the group without a hip fracture, a twice as high activity was expected. Assuming a similar variance, the power analysis resulted in 15 patients needed in the group without facture (>80% power, 2-sided confidence interval 95%).

For bivariate statistical analysis, data sets were tested for normal distribution, and then compared either by an unpaired student's *t*-test or a Mann–Whitney-*U*-test. Incidences were compared using the chi square test. Continuous variables assessed at the two different time points were compared using the paired student's *t*-test. The Spearman rho was calculated to analyze correlations. By this, possible confounding variables were identified and included in a multiple regression model. Age, gender, and BMI were considered as a priori risk factors for all outcomes and included in all model calculations. Model checks were carried out with each regression. The two-sided *p*-value was considered significant when <0.05.

## 3. Results

### 3.1. Participants' Characteristics

**Figure 1:**
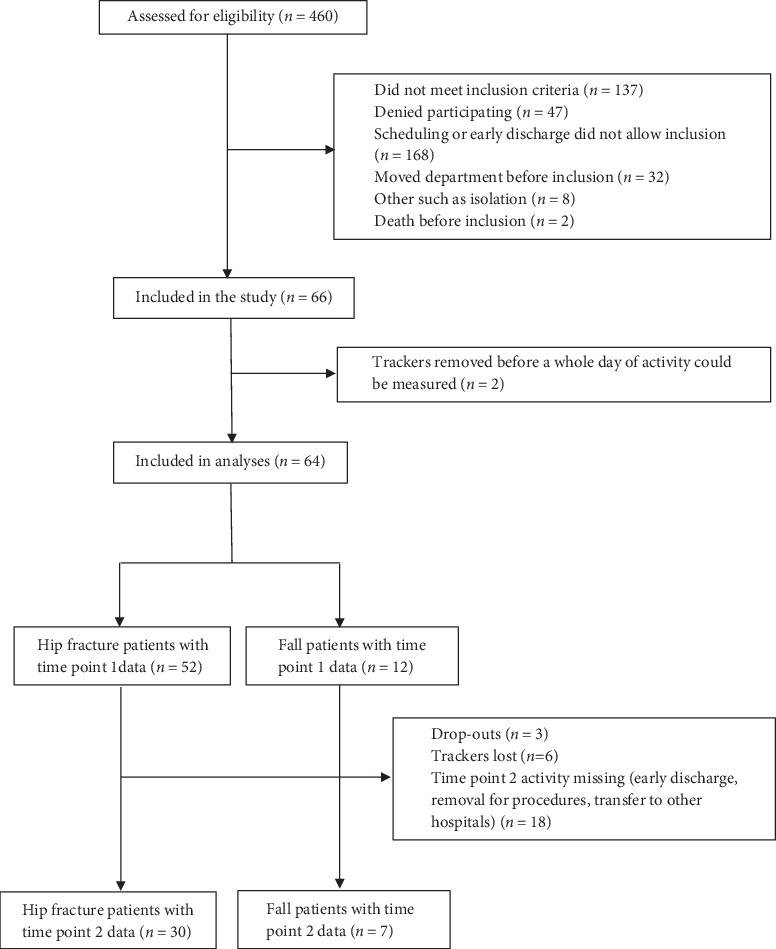
Flow chart inclusion.

While 66 participants were included in the present study, only 64 could be analyzed. 52 of them were part of the hip fracture group and 12 of the group without hip fracture, which nearly matches our prestudy power calculation. For 37 participants, activity data sets were acquired for both time points. Three trackers were lost in the mail and three were lost in the departments.  Figure 1 shows the flow chart for patient inclusion more detailed. The mean age of the total cohort was 81.2 years (SD 7.8), most were women (41/64), and the mean body mass index (BMI) was 24.07 (SD 4.7). The mean American Society of Anesthesiologists (ASA) Score was 2.41 (SD 0.56) indicating a high-risk population. Correspondingly, 25 of the patients with proximal femur fractures and 7 of the group without hip fracture suffered from complications. The average mobility status for the cohort reached 2.7 (SD 0.5), which means that most patients could walk without supporting devices before admission. This corresponded with a rather high preadmission Barthel-20 of 18.1 (SD 2.0).  Table 1 shows baseline characteristics and the comparison between the groups. Except CRP, no differences between the groups were observed.

In the hip fracture group, 31 patients were treated by an osteosynthesis and 21 by a bipolar hemiarthroplasty. 27 suffered from a femoral neck fracture, undergoing arthroplasty in most cases. All others had impacted valgus-fractures treated by screw fixation. 25 patients had an intertrochanteric fracture fixed by gamma nail (Stryker, Kalamazoo, MI, USA). All patients could bear full weight immediately after operation. The time from admission to operation was 27 hours (SD 46) in average, however, the median was 19 hours and the 75^th^ percentile 25 hours.

### 3.2. Change in Activity with Time

Comparing the values assessed at the different time points, the periods of “high activity,” active minutes, very active minutes, and the relative portion of very active minutes per day were higher before admission (time point 2) reaching statistical significance (Table 2). Besides these activity parameters, Barthel-20 and EQ5D-3L scores increased significantly from TP1 to TP2 in the total cohort. Principally, these changes were also observed in the investigated subgroups, however, caused by the lower case numbers these differences did not reach a statistical significance.

### 3.3. Parameters Influencing Activity

A multiple regression analysis assessed the influence of different conditions referring to the prefall conditions of included patients on the number of active minutes per day at time point 1 and time point 2. While age, CRP, and affirmation of the STS question were negatively associated with postoperative activity, a high prefall mobility, Barthel-20, BMI, and Albumin had a significant positive effect (Table 3). In this population with a normal weight (BMI < 25), the BMI positively correlated with the number of active minutes per day at time point 1. The admission albumin serum level also positively correlated with the number of active minutes per day at time point 1. Contrarily, a higher baseline CRP, indicating acute infection, correlated negatively with the active minutes. The other analyzed parameters ASA score, gender, and VRS for pain did not correlate with any of the assessed activity measures.

### 3.4. Correlation of Activity Measurement with Barthel-20 and EQ5D-3L

A multiple regression analysis was used to analyze the associations of the Barthel-20 index, and the EQ5D-3L (gold standard) with our assessed activity measures. Based on the calculations using the number of very active minutes per day, the results are exemplified in Table 4. A statistically significant association of Barthel-20 and EQ5D with very active minutes per day could be demonstrated at both investigated time points.

### 3.5. Prediction of the Patients' Recovery Process

Since activity measurement is thought to be used as predictor for the clinical progress during the hospital stay, the assessed activity measures at TP 1 were correlated with the golden standards Barthel-20 and EQ5D-3L, and with the activity measures at TP 2. The regression analysis revealed a highly statistically significant correlation for all parameters (Table 5). The quality of prediction for any kind of activity measurement was better than for the scores evaluating functional independence or quality of life. However, the activity measures did not correlate with the incidence or occurrence of complications. Neither of the assessed laboratory parameters assessed at admission or TP 1 could predict future activity.

### 3.6. Differences Between Groups with or without a Hip Fracture

There was no difference regarding any of the investigated activity parameters, Barthel-20, EQ-5D-3L or complications (number or just yes/no) between the two groups. The original values are referenced in Table 2. However, the difference of Barthel-20 measured at TP 1 and 2, reflecting the improvement during the hospital treatment, was significantly higher in the fracture group (5.26 ± 2.86 vs. 2.50 ± 3.62, *p* = 0.022). The difference for EQ5D showed the same phenomenon (0.34 ± 0.35 vs. 0.06 ± 0.21, *p* = 0.035), indicating that improvement during the hospital stay was better in the fracture group.

## 4. Discussion

The main findings of the study comprise of positive indicators for a high postoperative physical activity in patients with proximal femur fractures such as a high Barthel-20 index before fracture, a negative Sit-to-Stand index question, a low age, a high body mass index, high baseline albumin levels, and low C-reactive protein levels at admission. Accelerometer signals correlated significantly with EuroQol-5D and Barthel-20, scoring quality of life and functional independence. Objectively measured activity parameters predicted convalescence up to discharge just as reliably as these scores. Hereby, no difference was found comparing patients with or without a hip fracture. However, the recovery, as measured by increase in Barthel-20 and EQ5D scores, was significantly better among the orthopedic fracture patients compared to the admitted geriatric population. The significantly higher CRP in the fall group without a hip fracture is most likely caused by the fact that the admission was not only indicated by the fall but also by inflammatory diseases such as pneumonia or infection of the urinary bladder.

Although patients operated for proximal femur fractures move differently and walk slowly compared to a young and healthy population [28], their physical activity could be measured reliably using a specific algorithm developed for this purpose [20]. Hereby, the very low activity levels found in this cohort was consistent with earlier findings [10, 18, 29–31], although the absolute values were higher in this study caused by the recently adapted threshold [20]. The need for these adjusted algorithms and appropriate tracker devices is in accordance with studies, showing distinctive validity for different wearables and intensities [32, 33]. The clinical improvement observed between operation and discharge could be documented with a similar quality as the usually applied indices for quality of life (EQ5D) and functional independence (Barthel-20), which are regarded as the current gold standard [34]. However, only the measured activity parameters of the highest level out of 4 corresponding with actual walking or active or very active minutes were able to delineate this development. Therefore, activity tracking may not only be considered as a good alternative to these classical instruments but has the advantage of being an objective tool without the need for active collaboration of the patient. This has the potential to save nursing resources in future and allows a direct link to patients' medical records. Furthermore, potential cognitive deficits in this group are not an obstacle for monitoring PA by accelerometers. However, physical activity is a different construct compared to functional capacity and quality of life, making a complete substitution not reasonable, even if they are correlated. However, driven by similar needs for objective surveillance on geriatric wards [18], accelerometers have been used in other studies before, showing advantages for monitoring the acute rehabilitation process. Technically, other studies used multiple accelerometers to determine posture and movement, which seems complicated in a clinical setting [8]. Furthermore, the research grade ActiGraph GTX-3, a three-axis accelerometer [10, 30], was used for research purposes. Since it is usually attached to patients using an elastic belt at the hip, there are incision-related limitations in a cohort of hip fracture patients. Another applied opportunity is to use single-axis accelerometers such as ActivPAL, equaling activity with an upright position [18, 29, 31], which however does not allow to discriminate intensity levels and requires a precise and consistent attachment of the device. This appears plausible with the background of our data, showing that only the intense level reflected progress during treatment. However, the method applied in this study is similar effective in older people and provides a larger flexibility for possible extended applications and more active patients. Recent technical developments regarding ActivPAL allowed to measure signals in 3 dimensions [35].

The identified factors, predicting postoperative activity, consisted basically of the prefall physical conditions and nutrition status. There are actually numerous studies describing the importance of the Barthel index and its predictive value [36, 37]. Interestingly, a similar conclusion could be drawn by the simple Sit-to-Stand index question, which was recently evaluated for scoring sarcopenia [38]. Both indices depend on age, consequently, physical activity was also in the evaluated cohort decreasing with increasing age. Sarcopenia was recently linked to nutrition status [39] and outcome measures following proximal femur fractures [40], and therefore were included in this analysis. Although the questions selected from the Mini Nutritional Assessment were not associated with postoperative activity, BMI had a positive regression coefficient in our model. The patients of our cohort were in average not obese. Therefore, this finding supports the conclusion that a low BMI is associated with less activity. In elderly, low BMI indicates less physiologic and functional reserve from smaller muscle mass [41]. This explains the relationship between low BMI and less activity. However, it does not indicate that obesity correlates with high activity, because patients included had a BMI < 30. Besides this, preoperative serum albumin levels were determined as an independent risk factor for diminished postoperative activity and delayed recovery, correlating with the findings that malnutrition is a predictor for mortality after hip fractures [42] and preoperative mobility for long term survival [4, 43]. Considering this importance of physical activity in the recuperation process, it appears essential to recognize deficits as early as possible. Therefore, the relationship of activity parameters at time point 1 with outcome measures at time point 2 were examined, showing significant correlations not only for activity measures but also Barthel-20 and EQ5D. This might help to allocate rehabilitation resources early according to patient's needs. However, complications could not be predicted by any of the assessed tools, pointing out the necessity for a continuous clinical evaluation regardless of activity level.

Initially, a fall associated with a proximal femur fracture was supposed to be more limiting for activity than a fall without a fracture of the lower extremities. Therefore, the power calculation was done assuming a twice as high activity for patients without a fracture, because walking was supposed to be more impaired in case of a hip fracture. However, no differences were found between these two groups comparing activity, quality of life, functional independence, and complications during hospital treatment at all-time points. The starting conditions were very similar, only differing for the admission CRP levels, which were higher in the geriatric patient group without hip fracture. This indicates a higher portion of ongoing infectious diseases in the control cohort. Since high CRP levels were an independent risk factor for impaired physical activity, this obviously had a negative impact on activity of the patients without lower extremity fractures. Moreover, the data show that hip fracture patients can quickly increase their mobility, when a timely and correct operation is provided. Interestingly, the dynamics of recovery analyzed as differences between the time points after operation and before discharge were significantly higher in the hip fracture group. This indicates an unexpected higher degree of frailty among geriatric patients compared to the orthopedic cohort. This is a limitation in our study, because the a priori power calculation assumed that the fall patients would have a higher activity than hip fracture patient.

Considering the initial power calculation, the study is underpowered especially regarding the group without a hip fracture. However, the initial assumptions could not be confirmed. Other limitations of the study include a risk of recall bias regarding the prefall status, especially in this older population. To reduce this possible error, the OMC was assessed, evaluating mental skills, and included as an exclusion criterion. Generally, the environment of a hospital distracted many of the patients, which made it difficult to keep the patients focused. Therefore, it was important to take time for the interviews, making it as convenient and understandable as possible by i.e. reading questions loudly.

Considering the legal demand for an operation within 24 hours, the 26.9 hours mean time from injury to treatment reflects the inclusion of patients being treated with medical anticoagulants, requiring pausing medication, as well as patients who were admitted into hospital long after their fall.

According to our prestudy power analysis, 48 hip fracture patients had to enter the trial. Although even more were included, time point 2 data could not be acquired for all participants. This was predominantly due to early discharge, providing a good mobility, which was scored by physiotherapists and is a standardized discharge criterion. Therefore, missing data at time point 2 are related to more healthy and active patients, which were found equally in both cohorts.

## 5. Conclusions

It may finally be concluded that accelerometer signals reliably reflect postoperative physical activity in older patients, which is positively influenced by a high prefall functional independence and a good nutrition status. Prerequisites are suitable devices such as the used Axivity AX3 tracker and algorithms adapted to the low activity intensity of older patients following hospital admission. A timely and adequate operation provided, there is no difference between patients with or without hip fractures after low-energy trauma.

## Figures and Tables

**Table 1:** Baseline characteristics.

	All		Hip fracture group		Fall group		*P*
	*N*		*N*		*N*		
*Demographic*							
Age (mean ± SD)	64	81.2 ± 7.8	52	81.29 ± 7.45	12	80.83 ± 9.54	0.86
Gender (male/female)	64	23/41	52	16/36	12	7/5	0.1
BMI (mean ± SD)	62	24.1 ± 4.7	51	24.3 ± 4.45	11	23.04 ± 5.63	0.42

*Function/Health*							
ASA (mean ± SD)	64	2.4 ± 0.6	52	2.38 ± 0.53	12	2.5 ± 0.67	0.72
Mobility status (mean ± SD)	63	2.7 ± 0.5	51	2.67 ± 0.48	12	2.67 ± 0.49	1
STS (no/yes)	48	18/30	38	15/23	10	3/7	0.62
Barthel-20 (mean ± SD)	48	18.1 ± 2.0	38	18.26 ± 1.91	10	17.2 ± 2.25	0.14

*Treatment*							
Time from injury to treatment (mean ± SD)	64	27.03 ± 42.05	52	26.87 ± 45.71	12	27.75 ± 20.96	0.33
Length of stay (mean ± SD)	62	7.16 ± 3.13	50	7.28 ± 3.03	12	6.67 ± 3.63	0.55

*Laboratory parameters at admission*							
Hemoglobin (mmol/l)	61	7.56 ± 1.29	50	7.65 ± 1.19	11	7.15 ± 1.17	0.22
Albumin (g/l)	53	37.06 ± 5.09	42	37.48 ± 4.7	11	35.45 ± 6.36	0.24
C-reactive Protein (mg/l)	59	23.61 ± 38.07	48	18.73 ± 33.35	11	44.91 ± 50.66	0.008^∗^

*Nutrition*							
Current weight loss—6 months (no/yes)	48	28/20	38	25/13	10	3/7	0.07
Eating reduction—1 week (no/yes)	48	31/17	38	26/12	10	5/5	0.3

The table shows baseline features for the whole cohort, patients with a proximal femur fracture and patients with a fall without fracture. The parameters are grouped for patients' demographic characteristics, the prefall function and health status, treatment quality, baseline laboratory parameters, and nutrition status. The last column shows the *p*-value for the difference between the fracture group and the fall group. BMI: body mass index. ASA: American Society of Anesthesiologists Score. STS: “sit-to-stand” index. *N*: number of participants in analysis. SD: standard deviation. ^∗^Indicates statistical significance.

**Table 2:** Increase of activity in the different groups with time.

Category	All			Hip fracture group			Fall group		
	TP1	TP2	*P*	TP1	TP2	*P*-value	TP1	TP2	*P*
	Mean ± SD	Mean ± SD		Mean ± SD	Mean ± SD		Mean ± SD	Mean ± SD	
Periods of “High activity”	13.85 ± 12.28	18.44 ± 17.76	0.048^∗^	14.34 ± 12.33	18.72 ± 17.46	0.09	11.7 ± 12.48	17.26 ± 20.40	0.29
Active minutes	248.92 ± 154.59	309.02 ± 200.67	0.049^∗^	257.11 ± 145.98	311.9 ± 188.08	0.108	213.7 ± 192.17	296.67 ± 265.38	0.15
Very active minutes	90.01 ± 66.97	122.87 ± 94.89	0.002^∗^	89.44 ± 62.19	123.39 ± 84.7	0.006^∗^	92.45 ± 88.67	120.65 ± 139.07	0.23
Percent very active minutes	6.25 ± 4.65	8.61 ± 6.54	0.002^∗^	6.21 ± 4.32	8.66 ± 5.81	0.005^∗^	6.42 ± 6.16	8.38 ± 9.66	0.23
Bathel-20	8.55 ± 3.3	12.81 ± 3.5	<0.001^∗^	8.24 ± 2.92	13.03 ± 3.06	<0.001^∗^	10.1 ± 4.68	11.75 ± 5.28	0.09
EQ5D-3L	0.27 ± 0.37	0.52 ± 0.28	<0.001^∗^	0.27 ± 0.37	0.56 ± 0.22	<0.001^∗^	0.29 ± 0.36	0.28 ± 0.41	0.48

This table shows the relevant activity outcomes, demonstrating a significant increase from time point 1 to time point 2 for the total cohort. Furthermore, this difference was analyzed in the subgroups. TP: time point. EQ5D-3L: European quality of life 5 dimension 3 levels. SD: standard deviation. ^∗^Indicates statistically significant difference between time point 1 and time point 2 for each group.

**Table 3:** Regression analysis for parameters with influence on active minutes.

	Time point 1			Time point 2		
	Coefficient	*P*-value	*R* ^2^	Coefficient	*P*-value	*R* ^2^
Age	−6.67	0.021	0.18	−9.35	0.037	0.19
Premobility	90.14	0.036	0.25	168.51	0.007	0.36
STS	−132.34	0.010	0.38	−162.15	0.036	0.43
Preadmission Barthel-20	35.2	0.012	0.38	45.92	0.029	0.43
BMI	11.51	0.026	0.38	n.s.		
CRP	−1.6	0.028	0.25	n.s.		
Albumin	2.95	0.044	0.24	n.s.		

This table shows the results of the multiple regression analysis for the influential parameters on active minutes at different time points. For the regression with the laboratory parameters, admission values were used. STS: “sit-to-stand” index. BMI: body mass index. CRP: C-reactive protein. n.s.: not statistically significant.

**Table 4:** Correlations of activity measures with Barthel-20 and EQ5D-3L scores.

	Time point 1			Time point 2		
	Coefficient	*P*-value	*R* ^2^	Coefficient	*P*-value	*R* ^2^
Barthel-20	11.03	<0.001	0.34	15.09	0.002	0.41
EQ5D-3L	85.23	0.001	0.30	124.43	0.016	0.30

This table shows the results of the regression analysis analyzing the correlation of very active minutes per day with the Barthel-20 and EQ5D-3L scores. Time point 1 and 2 indicate associations of activity with the outcome scores at the respective time points. EQ5D-3L: European quality of life 5 dimensions 3 levels.

**Table 5:** Prediction of outcomes during the hospital stay.

	Coefficient	*P*-value	*R* ^2^
Periods of high activity	0.07	<0.001	0.50
Active minutes	0.79	<0.001	0.50
Very active minutes	0.36	<0.001	0.50
Barthel-20	11.03	<0.001	0.34
EQ5D-3L	85.23	0.001	0.30
Complications	n.s.		

This table shows the results of the regression analysis with the different outcome measures at time point 2 and the active minutes at time point 1. EQ5D-3L: European quality of life 5 dimensions 3 levels. n.s.: not statistically significant.

## Data Availability

The datasets generated and/or analyzed during the current study are not publicly available due data safety considerations but are available from the corresponding author on reasonable request when following EU General Data Protection Regulations.

## Abbreviations

ASA: American society of anesthesiologists
AO: Arbeitsgemeinschaft für osteosynthesefragen (Association for osteosynthesis issues)
BMI: Body mass index
CRP: C-reactive protein
EQ5D-3L: European quality of life 5 dimensions 3 levels
HGB: Hemoglobin
OMC: Orientation memory concentration
OUH: Odense university hospital
PA: Physical activity
SD: Standard deviation
STS: Sit-to-stand
TP: Time point
VRS: Verbal rating scale.
